# Predicting Positive ELISA Results in Dairy Herds with a Preferred Status in a Paratuberculosis Control Program

**DOI:** 10.3390/ani12030384

**Published:** 2022-02-04

**Authors:** Maarten F. Weber, Marian Aalberts, Thomas Dijkstra, Ynte H. Schukken

**Affiliations:** 1 Royal GD, P.O. Box 9, 7400 AA Deventer, The Netherlands; m.aalberts@gdanimalhealth.com (M.A.); t.dijkstra@gdanimalhealth.com (T.D.); y.schukken@gdanimalhealth.com (Y.H.S.); 2 Department of Population Health Sciences, Faculty of Veterinary Medicine, Utrecht University, Yalelaan 7, 3584 CL Utrecht, The Netherlands; 3 Quantitative Veterinary Epidemiology, Department of Animal Sciences, Wageningen University, P.O. Box 338, 6700 AH Wageningen, The Netherlands

**Keywords:** paratuberculosis, dairy cattle, control program, predictive model

## Abstract

**Simple Summary:**

In many paratuberculosis control programs, test-negative herds are assigned a preferred herd status. This may induce the false belief that these herds are free of paratuberculosis. Hence, farmers may refrain from control measures that could prevent the spread of any undetected infections. The aim of the present study was to develop a predictive model to alert farmers with test-negative herds and a preferred status in the Dutch paratuberculosis control program if they are at an increased risk of positive ELISA results in a subsequent 30-month period. On the basis of the results of this study, we conclude that discrimination of herds with high (52%) and low (17%) risks of positive ELISA results is feasible. This might help farmers with the highest risks of future positive results to make better informed decisions regarding the need to take additional control measures to prevent the spread of any undetected Map infections.

**Abstract:**

Dairy herds participating in the Dutch milk quality assurance program for paratuberculosis are assigned a herd status on the basis of herd examinations by ELISA of individual serum or milk samples, followed by an optional confirmatory fecal PCR. Test-negative herds are assigned Status A; the surveillance of these herds consists of biennial herd examinations. Farmers falsely believing that their Status A herds are Map-free may inadvertently refrain from preventive measures. Therefore, we aimed to develop a predictive model to alert Status A farmers at increased risk of future positive ELISA results. Using data of 8566 dairy herds with Status A in January 2016, two logistic regression models were built, with the probabilities of ≥1 or ≥2 positive samples from January 2017–June 2019 as dependent variables, and province, soil type, herd size, proportion of cattle born elsewhere, time since previous positive ELISA results, and the 95th percentile of the S/P ratios in 2015–2016, as explanatory variables. As internal validation, both models were applied to predict positive ELISA results from January 2019–June 2021, in 8026 herds with Status A in January 2019. The model predicting ≥1 positive sample had an area under the receiver operating characteristics curve of 0.76 (95% CI: 0.75, 0.77). At a cut-off predicted probability π_c_ = 0.40, 25% of Status A herds would be alerted with positive and negative predictive values of 0.52 and 0.83, respectively. The model predicting ≥2 positive samples had lower positive, but higher negative, predictive values. This study indicates that discrimination of Status A herds with high and low risks of future positive ELISA results is feasible. This might stimulate farmers with the highest risks to take additional measures to control any undetected Map infections.

## 1. Introduction

Paratuberculosis is an inflammatory bowel disease, primarily affecting ruminants, that is caused by *Mycobacterium avium* subsp. *paratuberculosis* (MAP). Paratuberculosis is of concern to dairy cattle industries worldwide because of its economic impact on dairy production [1,2] and uncertainty about the potential involvement of MAP in human disease [3,4]. Control programs for paratuberculosis have been developed in at least 22 countries [5]. In several programs for paratuberculosis, test-negative herds or herds with a high probability of absence of the infection are assigned an assurance score or preferred herd status [5,6].

In 2006, a milk quality assurance program (MQAP) for paratuberculosis in Dutch dairy herds was initiated. The aim of this MQAP is to reduce the concentration of Map in milk delivered to the milk processing industry by controlling Map in the participating herds [6,7,8]. Herds participating in the MQAP are assigned a herd status (A, B, or C) on the basis of the results of herd examinations [9]. In short, an initial assessment of the participating herds consists of a single herd examination. Test-negative herds enter a surveillance procedure and are assigned Status A. The surveillance of herds with Status A consists of biennial herd examinations. Test-positive herds at the initial assessment or during the surveillance procedure, enter a control procedure and are assigned Status B (if all test-positive cattle have been removed from the herd) or Status C (if any test-positive cattle are retained in the herd). If an annual herd examination in a herd with Status B yields negative results only, then the herd progresses to Status A. Each herd examination consists of either testing individual milk samples of all lactating cattle, or serum samples of all cattle ≥3 years of age, by an ELISA for antibodies against Map. The confirmation of positive ELISA results by individual fecal PCR assay or culture is optional. 

The test scheme of the MQAP was developed to provide assurance that herds with Status A have a high probability of either the absence, or a low concentration, of Map in the milk (<10^3^ organisms per liter) delivered to the milk processors [7,8,10]. This does not necessarily mean that a Status A herd is Map-free. Thus, the program can be run at considerably lower costs than a program aiming to certify herds as Map-free [8,11]. However, undetected infections may contribute to the rather high rate of loss of Status A that has been observed [12]. Herds that were assigned Status A at the initial assessment had a probability of 43% of losing this status within ten years after the initial assessment. Herds that were assigned Status B or Status C at the initial assessment and progressed to Status A at a later stage, subsequently had a probability of 81% of losing Status A within eight years [12].

Preventive management measures may reduce the risk that undetected Map infections in Status A herds result in the loss of status A. However, many farmers with Status A herds indicate, in personal contacts, that they refrain from taking preventive management measures on the basis of their belief that their herds are Map-free. Therefore, the aim of the present study was to develop a predictive model to alert farmers with Status A herds if they are at an increased risk of positive ELISA results.

## 2. Materials and Methods

### 2.1. Laboratory Assays

For the participating herds, the results of serum samples and individual milk samples submitted from January 2006 onwards were retrieved from the laboratory information system of Royal GD. The individual milk samples and serum samples were tested by commercial ELISAs at varying cut-off values (Table 1). However, for the development and validation of the predictive model, a cut-off value of 1.0 was used, irrespective of the sample type (milk or serum), the ELISA kit (A or B), or the date of submission of the sample. The cut-off value in the present study was chosen in line with the cut-off of 1.0 used in the MQAP for milk samples tested with ELISA Kit A (Table 1), which constituted the majority of samples included in the training dataset (see below, Section 3.1). Thus, for the purpose of this study, all valid ELISA results with a sample-to-positive ratio (S/P) <1.0 were considered negative, and all ELISA results with an S/P ≥1.0 were considered positive.

### 2.2. Development of the Predictive Model

To explore whether a high risk of loss of the preferred herd status (Status A) could be adequately predicted, a logistic regression analysis was performed on data of herds that had Status A on 1 January 2016. The primary outcome of interest was whether the herd had at least one ELISA result with an S/P ≥1.0 of a sample submitted in a 30-month period between 1 January 2016 and 30 June 2018. This 30-month frame was chosen to ensure that at least one herd examination was performed in each herd, given that the interval between the herd examinations in Status A herds is approximately 24 months. The secondary outcome of interest was whether the herd had at least two samples with an S/P ≥1.0 submitted in the same 30-month period. These outcome variables were chosen because, other than the loss of Status A, they are independent of farmers’ decisions on confirmatory fecal testing of cattle with positive ELISA results.

The retrospective data collection and analyses to develop the model were restricted to all herds that matched the following selection criteria: (a) The initial assessment of the herd was prior to 1 January 2011; (b) The herd had a bulk milk tank number and was participating in the MQAP on 1 January 2014, 1 January 2016, and 1 July 2018; (c) The herd was assigned Status A by 1 January 2016; (d) ≥50 ELISA results of samples submitted from the herd between 1 January 2014 and 31 December 2015; (e) On 1 January 2016, the herd had at least 50 adult cattle (≥2 years of age); (f) The S/P could be retrieved for at least one ELISA result of samples submitted from the herd between 1 January 2016 and 30 June 2018; (g) The predominant soil type of the postal code area of the herd could be retrieved.

Putative explanatory variables that were included in the full logistic regression model were: the province of the Netherlands; the predominant soil type in the upper 80-cm layer of soil in the terrestrial surface area of the 4-digit postal code of the herd (sand, bog, sandy loam, clay, or other); the herd size (number of cattle ≥2 years of age on 1 January 2016); the proportion of cattle present in the herd on 1 January 2016 that were born in another herd; the time elapsed since the last ELISA result with an S/P ≥1.0; and the 95th percentile of the distribution of the S/P ratios between 1 January 2014 and 31 December 2015. The 95th percentile of the S/P ratios was chosen because, in comparison with the 1st, 5th, 10th, 25th, 50th, 75th, and 90th percentiles, preliminary univariable receiver-operating characteristics (ROC) analyses indicated a higher area under the curve for prediction of at least one future positive ELISA result based on the 95th percentile. Furthermore, the 95th percentile had fewer missing values than the 99th percentile, as the 99th percentile is not meaningful in herds with less than 100 samples tested. To avoid overfitting, none of the interactions between the explanatory variables were tested. Preliminary analyses indicated nonlinearity of the effects of the time elapsed since the last ELISA result with an S/P ≥1.0 and the proportion of cattle that were born in another herd. Therefore, these variables were categorized (Table 1). Multicollinearity between the putative explanatory variables was evaluated by linear regression [13]. A tolerance <0.25 and a variance inflation factor >4 were considered indicative of multicollinearity.

For both outcomes of interest, a final model was obtained in a backward elimination procedure, with a threshold probability for stepwise removal of 0.10 in the likelihood ratio test. Confounding was monitored by the changes in the regression coefficients. If elimination of a variable resulted in a change in the estimated regression coefficient of any other variable exceeding 25% or 0.1, in case of an estimate between −0.4 and 0.4, the eliminated variable was considered a potential confounder and was re-entered into the model. The model fit was evaluated with the Hosmer–Lemeshow goodness-of-fit test. To correct for overfitting, the model parameters were multiplied with a heuristic shrinkage factor [14]:

(1) $$SF_{heuristic}=\frac{\left(\mathrm{model}\;\chi^{2}-\mathrm{df}\right)}{\mathrm{model}\;\chi^{2}}$$

after which the intercept was calibrated to align the average predicted probability of at least one (primary outcome of interest) or two (secondary outcome of interest) samples with an S/P ≥1.0 between 1 January 2016 and 30 June 2018, with the observed probabilities. The predicted probability $\pi_{i}$ of herd *i* to have at least one or two samples with an S/P ≥1.0 was calculated on the basis of the point estimates of the model coefficients. If the logistic regression model reads:

(2) $$\mathrm{logit}\left(\pi \right)=\ln\left(\frac{\pi }{1-\pi }\right)=\beta_{0}+\beta_{1}X_{1}+\beta_{2}X_{2}+\cdots +\beta_{n}X_{n}$$

then the $\pi_{i}$ of herd *i* is calculated as:

(3) $$\pi_{i}={\left[1+e^{-\left(\beta_{0,\;adjusted}+SF_{heuristic}\left(\beta_{1}X_{1}+\beta_{2}X_{2}+\cdots +\beta_{n}X_{n}\right)\;\right)}\right]}^{-1}$$

in which $\beta_{0,\;adjusted}$ is the calibrated intercept, and $\beta_{1},\;\beta_{2},\;\ldots ,\;\beta_{n}$ are the coefficients of the explanatory variables $X_{1},\;X_{2},\;\ldots ,\;X_{n}$ in the logistic regression model. Categorical explanatory variables were included in the prediction model as dummies for the various levels of each variable, with the exception of the reference categories of the variables. The dummy variables were coded as 1 if applicable (true), and 0 if not applicable (false), for herd *i*. The performances of the predictive models were evaluated in ROC analyses on the predicted probabilities of at least one (primary outcome of interest) or two (secondary outcome of interest) samples with an S/P ≥1.0, in comparison with the observed outcome in each herd. In the ROC analyses, the proportion of herds considered to be at an increased risk of positive ELISA results at various cut-off values $\pi_{c}$, as well as the sensitivity, specificity, and positive and negative predictive values of the prediction model at this cut-off value, were calculated by comparing the $\pi_{i}$ of each herd in the dataset with the cut-off value $\pi_{c}$.

### 2.3. Internal Validation of the Predictive Models

As an internal validation, the predictive models were applied to a second dataset of herds with Status A on 1 January 2019, predicting whether the herd had at least one (primary outcome of interest) or two (secondary outcome of interest) ELISA results with an S/P ≥1.0 of a sample submitted in a 30-month period between 1 January 2019 and 30 June 2021.

The dataset used for this internal validation consisted of all herds that matched the following selection criteria: (a) The initial assessment of the herd was prior to 1 January 2013; (b) The herd had a bulk milk tank number and was participating in the MQAP on 1 January 2017, 1 January 2019, and 1 July 2021; (c) The herd was assigned Status A by 1 January 2019; (d) ≥50 ELISA results of the samples submitted between 1 January 2017 and 31 December 2018; (e) On 1 January 2019, the herd had at least 50 adult cattle (≥2 years of age); (f) The S/P could be retrieved for at least one ELISA result of samples submitted from the herd between 1 January 2019 and 30 June 2021; (g) The predominant soil type of the postal code area of the herd could be retrieved.

The data on the explanatory variables that were included in the predictive models were retrieved in a similar manner as in the development phase, with 1 January 2019 as a reference date. The province of the Netherlands, the predominant soil type, the herd size (number of cattle ≥2 years of age), the proportion of cattle present in the herd that were born in another herd, and the time elapsed since the last ELISA result with an S/P ≥1.0 were determined on 1 January 2019. The 95th percentile of the S/P ratios was calculated using ELISA results of samples submitted between 1 January 2017 and 31 December 2018.

Finally, the performance of the predictive models was evaluated in ROC analyses on the predicted probabilities of at least one (primary outcome of interest) or two (secondary outcome of interest) samples with an S/P ≥1.0, in comparison with the observed outcome in each herd.

## 3. Results

### 3.1. Development of the Predictive Models

**Figure 1:**
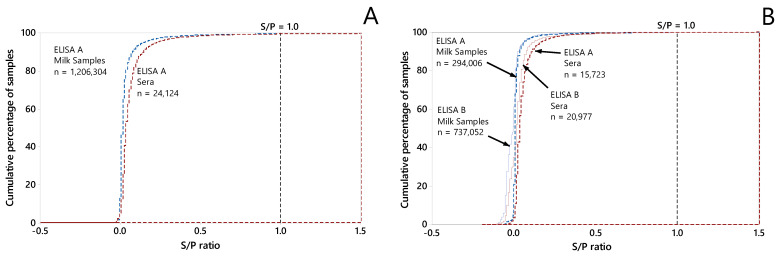
Cumulative distributions of S/P ratios of serum and milk samples included in the outcome variable. (**A**) Training dataset: samples submitted between 1 January 2016 and 30 June 2018 from 8566 Dutch dairy herds. (**B**) Validation dataset: samples submitted between 1 January 2018 and 30 June 2021 from 8026 Dutch dairy herds.

A total of 8566 dairy herds with Status A on 1 January 2016 matched all the selection criteria for the training dataset. Between 1 January 2016 and 30 June 2018, 1,230,699 samples submitted from these herds were tested by ELISA. For 271 of these samples, the S/P could not be retrieved. The distribution of the S/P of the remaining 1,230,428 samples is shown in Figure 1. Only 5282 (0.4%) of the tested samples had an S/P ≥1.0. These 5282 samples with an S/P ≥1.0 were clustered in 2347 (27%) of the 8566 dairy herds. In those 2347 herds, the mean proportion of samples with an S/P ≥1.0 was 1.3% (min: 0.07%; max: 13.2%). From 1126 (13%) of the 8566 herds, at least two samples with an S/P ≥1.0 were submitted.

All explanatory variables that were entered in the full logistic regression models were retained in the final logistic regression models (Table 2 and Table 3). The numbers of adult cattle in the 8566 herds on 1 January 2016 ranged from 50 to 874 (median: 103; mean: 115) cattle. The 95th percentile of the S/P ratios between 1 January 2014 and 31 December 2015 ranged from 0.02 to 1.60 (median: 0.13; mean: 0.16). In both analyses, the *SF_heuristic_* equalled 0.98 and, consequently, the adjusted beta values in the predictive models only marginally differed from the estimated beta values in the final logistic regression models (Table 2 and Table 3).

**Figure 2:**
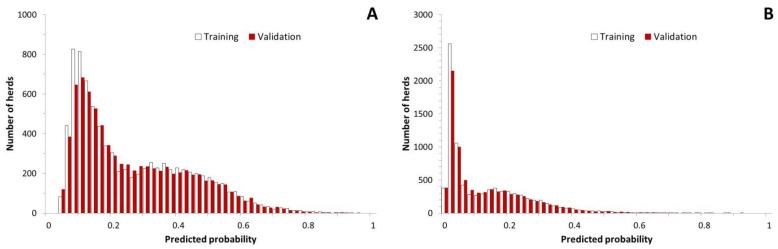
Distribution of predicted probabilities of (**A**) at least one, and (**B**) at least two ELISA result(s) with an S/P ≥1.0 in the training dataset (8566 dairy herds with Status A on 1 January 2016) and the validation dataset (8026 dairy herds with Status A on 1 January 2019).

**Figure 3:**
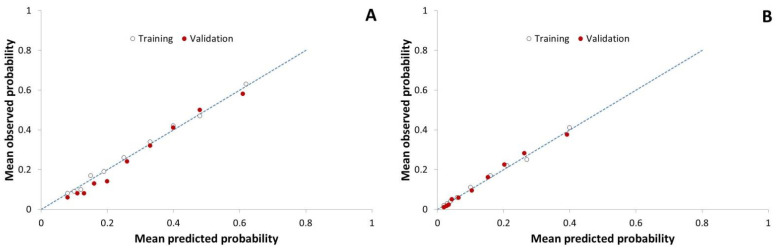
Mean observed probabilities and mean predicted probabilities of (**A**) at least one, and (**B**) at least two ELISA result(s) with S/P ≥1.0 for observations in each of the ten deciles of the predicted probabilities in the training dataset (8566 dairy herds with Status A on 1 January 2016) and the validation dataset (8026 dairy herds with Status A on 1 January 2019). The dashed lines represent the identity line where the mean observed probability equals the mean predicted probability.

**Figure 4:**
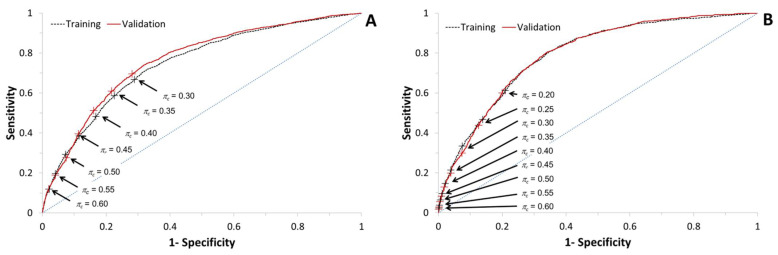
Receiver-operating characteristics curve of the predictive models in the training dataset (8566 dairy herds with Status A in the Dutch milk quality assurance program for paratuberculosis on 1 January 2016) and the validation dataset (8026 dairy herds with Status A in the Dutch milk quality assurance program for paratuberculosis on 1 January 2016). (**A**) Predictive model for at least one ELISA result with an S/P ≥1.0. (**B**) Predictive model for at least two ELISA results with an S/P ≥1.0. Arrows and + signs indicate various cut-off values *π_c_* of the predicted probability *π* of at least one or at least two ELISA result(s) with an S/P ≥1.0 on the ROC curves. The dashed lines represent the identity line.

With both outcomes of interest, the distributions of the predicted probabilities in the training set were skewed: there were few herds with a high (≥0.8) predicted probability of at least one ELISA result with an S/P ≥1.0 (Figure 2). However, the predicted probabilities of at least one (primary outcome of interest) or two (secondary outcome of interest) samples with an S/P ≥1.0 were close to the observed probabilities (Figure 3). In the training dataset, the predictive models for at least one (primary outcome of interest) or two (secondary outcome of interest) samples with an S/P ≥1.0 had areas under the ROC curve equal to 0.747 (95% CI: 0.735; 0.759) and 0.791 (0.778; 0.804), respectively (Figure 4).

### 3.2. Internal Validation

A total of 8026 dairy herds with Status A on 1 January 2019 matched all selection criteria for the validation dataset. Between 1 January 2019 and 30 June 2021, 1,068,046 samples submitted from these herds were tested by ELISA. For 288 of these samples, the S/P ratio could not be retrieved. The distribution of the S/P ratios of the remaining 1,067,758 samples is shown in Figure 1. Only 4764 (0.4%) of these samples had an S/P ≥1.0. These 4764 samples with an S/P ≥1.0 were clustered in 2036 (25%) of the 8026 dairy herds. In those 2036 herds, the mean proportion of samples with an S/P ≥1.0 was 1.3% (min: 0.08%; max: 50%). From 1041 (13%) of the 8026 herds, at least two samples with an S/P ≥1.0 were submitted.

The numbers of herds in the various levels of the predictive variables in the predictive model are shown in Table 2. The numbers of adult cattle in the 8026 herds on 1 January 2019 ranged from 50 to 762 (median: 97; mean: 110) cattle. The 95th percentile of the S/P ratios between 1 January 2017 and 31 December 2018 ranged from 0.00 to 1.12 (median: 0.14; mean: 0.17). The distribution of the predicted probabilities of at least one sample with an S/P ≥1.0 was right-skewed in the validation dataset as well (Figure 2). However, in contrast to the training set, in the validation dataset, the observed probabilities of at least one sample with an S/P ≥1.0 were slightly overestimated by the prediction model at the lower range of the predicted probabilities (<0.3; Figure 3A). In the validation dataset, the predictive models for at least one (primary outcome of interest) or two (secondary outcome of interest) samples with an S/P ≥1.0 had areas under the ROC curve equal to 0.762 (95% CI: 0.750; 0.774) and 0.792 (0.779; 0.806), respectively (Figure 4).

### 3.3. Comparison of Model Performances in the Training and Validation Datasets

For each of the two models, the areas under the ROC curves in the training and validation datasets were comparable. Depending on the desired characteristics of the predictive models, a cut-off predicted probability *π_c_* of at least one ELISA result with an S/P ≥1.0 can be chosen that corresponds to a data point on the ROC curves (Figure 4). The proportion of herds with a predicted probability exceeding the cut-off value, i.e., the proportion of herds that would receive an alert, and the positive and negative predictive values at the various cut-off values *π_c_* of the predicted probability *π* are shown in Table 4.

For each of the two predictive models, the results obtained differed only marginally between the training and validation datasets. For example, at *π_c_* = 0.40 in the predictive model for at least one sample with an S/P ≥1.0, 25% of the Status A herds would receive an alert in both datasets. Of the herds receiving an alert, 52% would indeed have at least one ELISA result with an S/P ≥1.0 in the next 30 months. Of the herds not receiving an alert, 81% (training set) to 83% (validation set) would receive no ELISA result with an S/P ≥1.0 in the next 30 months, while the remaining 17 to 19% would receive at least one ELISA result with an S/P ≥1.0 (Table 4).

### 3.4. Comparison of Performances of the Two Predictive Models

In both the training and validation sets, the predicted probability of a herd to have at least two positive ELISA results was always lower than the predicted probability of the herd to have at least one positive ELISA result. The area under the ROC curves (Figure 4) was higher for the model predicting ≥2 samples with an S/P ≥1.0, in comparison to the model predicting ≥1 sample with an S/P ≥1.0. At a given cut-off probability *π_c_*, the model predicting ≥2 samples with an S/P ≥1.0 resulted in a lower proportion of herds with a predicted probability above the cut-off, a lower sensitivity and positive predictive value, and a higher specificity and negative predictive value than the model predicting ≥1 sample with an S/P ≥1 (Figure 4, Table 4).

However, at similar proportions of herds with a predicted probability above the cut-off (e.g., a cut-off predicted probability of 0.40, corresponding to 25% of herds in the model predicting ≥1 sample with an S/P ≥1.0, and a cut-off predicted probability of 0.20, corresponding to 25 to 26% of herds in the model predicting ≥2 samples with an S/P ≥1.0), the model predicting ≥2 samples with an S/P ≥1.0 resulted in a lower positive predictive value and a higher negative predictive value than the model predicting ≥1 sample with an S/P ≥1.0 (Table 3).

## 4. Discussion

The aim of the present study was to develop a predictive model to alert farmers with Status A if they are at an increased risk of positive (S/P ≥1.0) ELISA results. The resulting predictive models were able to discriminate between herds with an increased risk (in the range of 31 to 67%, depending on the choice of model and cut-off, and shown as the positive predictive values in Table 4), and herds with a rather low risk (in the range of 7 to 25%, being the complement of the negative predictive values shown in Table 4). The results show that the performances of both models were remarkably stable in time, with the area under the ROC curves being even higher in the validation dataset than in the training dataset. Even though the model predicting ≥2 positive samples resulted in a slightly higher area under the ROC curve than the model predicting ≥1 positive sample, the choice between the two models should be primarily based on the required test characteristics. At similar proportions of herds with a predicted probability above the cut-off, the model predicting ≥1 positive sample resulted in a higher positive predictive value but a lower negative predictive value than the model predicting ≥2 samples. Because the aim of the present study was to identify herds with an increased risk of positive ELISA results (whether true or false positive), a higher positive predictive value would be preferred.

The predictive models were based on the data available in the central datasets and included, as explanatory variables, the province, soil type, number of adult cattle, proportion of cattle born elsewhere, time evolved since the last previous positive ELISA result and the 95th percentile of the S/P ratios of samples submitted in the previous 24 months. These explanatory variables were based on the results of previous studies, and their effects were considered biologically plausible. However, it is important that the outcome in the present study was not simply the presence or absence of Map infection, but, rather, test-positivity in herds that were previously test-negative and hence acquired the preferred herd Status A. Thus, effects observed in the present study may differ from the results of studies that focus on presence or absence of Map infection, or that focus on presence or absence of positive test results. 

In line with the results of the present study, regional differences of the paratuberculosis prevalence have been described previously in the Netherlands [15], as well as in other countries [16,17,18,19]. In this study, in comparison to the sand soil type, clay was found to be associated with a higher probability of positive results in both models, whereas bog was significantly associated with the probability of ≥1 sample, but not the probability of ≥2 samples, with an S/P ≥1.0. The effects of soil type on Map transmission may be confounded by effects on the Map transmission of farming practices that are associated with soil type. In previous studies, high soil iron content [20,21], low pH [16,21,22,23,24], low silt content [25], high silt content [19], high loam content [19], high clay content [19,20], and high organic carbon content [20] were associated with Map prevalence or the incidence of clinical paratuberculosis. Thus, the inclusion of soil type in our predictive models is coherent with pre-existing evidence.

Several biological pathways may contribute to the observed association between the number of adult cattle and the probability of samples with an S/P ≥1.0. Firstly, in random mixing conditions, the probability of infectious contacts between susceptible and infectious cattle increases with herd size, resulting in an increased transmission until an endemic equilibrium is reached [26,27], as well as in a reduced probability of fade- out of the infection [28]. Several field studies have observed a positive association between herd size and the incidence of paratuberculosis or the (apparent or true) prevalence of Map infection [19,29,30,31,32,33,34,35,36]. The effect of herd size may be partially counteracted by effects of herd size on herd management, given that larger herds more frequently have separate facilities for calf-rearing [37]. Secondly, the number of samples tested increases with herd size. Serum and milk ELISAs have imperfect sensitivities and specificities [38,39]. With an increasing number of samples tested, the probability of at least one false positive result as well as, in infected herds, the probability of at least one true positive result increase [40].

In a consensus statement of the International Association for Paratuberculosis, appropriately documenting the probability of the freedom from Map infection was considered an important aspect of herd classification programs [41]. In simulation modeling studies, this probability of freedom from Map depended on herd size, the number of introduced cattle, and the time during which only negative test results were obtained [40,42]. These results are in line with our observations of the effects of herd size, the proportion of introduced cattle, and the time since the last ELISA result with an S/P ≥1.0 on the probability of obtaining positive ELISA results.

Predictive modeling to predict the loss of a preferred herd status has been applied before, with varying success, to control programs for other infectious diseases, such as BVD and salmonellosis [43,44]. In their study on BVD, Gates et al. [43] suggested to annually test replacement heifers for BVD virus in order to reduce the effect of introduction of persistently infected heifers into the dairy herd. Likewise, the annual testing of nulli-parous heifers for antibodies against Map, or Map shedding, might enable the earlier detection of a Map infection in Status A herds, given that a considerable proportion of infected young stock become test-positive before adulthood [45,46,47].

In the present study, the proportion of cattle born in other herds was used as a proxy for potentially infectious contacts between herds through the introduction of cattle. Cattle purchases have been found to be a risk factor for Map infection in other studies as well [29,48,49]. However, the proportion of cattle born in other herds does not cover risks of other cattle movements between herds, such as rearing of young stock of multiple herds together in specialized young stock herds. No data on herd management were available, other than herd size and the proportion of cattle born in other herds. It is generally assumed that herd management has a major effect on the spread of Map. However, in both Danish and Minnesota dairy herds, herd management was found to be a poor predictor of paratuberculosis seroprevalence [48,50]. Presumably, there is a time lag of at least 4 to 5 years (a one-cow generation) before effects of changes in herd management become apparent [50]. Hence, in order to substantially improve our model predictions by adding data on (changes in) herd management, a long-term inventory of herd management in participating herds may be required, which may not be practically feasible. 

In this study, a cut-off S/P ratio of 1.0 was used in both the dependent and explanatory variables in the models, whereas in the MQAP various cut-off values between 0.9 and 1.0 were used, depending on the ELISA kit, sample type (milk or serum), and date of submission of the sample. However, few samples had an S/P between 0.9 and 1.1 (Figure 1). Therefore, the proportion of positive results was considered rather insensitive to the exact choice of the cut-off value in this range of S/P ratios. The cut-off S/P ratio was chosen for the Dutch MQAP to increase the specificity and positive predictive value of the ELISA [12]. An important argument for this was that the objective of the program was to reduce the milk burden of MAP [7,8,9,10,12]. The MQAP, therefore, particularly aimed to identify high shedders that are known to have relatively high ELISA S/P values. Obviously, this decision also resulted in a relative low sensitivity and negative predictive value of the test.

The median values of the 95th percentile of the S/P ratios in the training and validation datasets were 0.13 and 0.14, respectively. At the low range of S/P ratios, some batch-to-batch variation may be expected, as verification of new batches of an ELISA kit is mainly focused on test performance around the cut-off values. However, the observed stability of the performance of our predictive models over time indicates that the model performance was rather insensitive to such batch-to-batch variation.

Our models were developed with annual predictions at a single point in time for all Status A herds in mind, irrespective of the date of the last herd examination in these herds. This would allow all data to be processed once per year, including an update of the coefficients of the model variables based on the most recent available data, followed by application of the model to predict which of the herds with Status A at the time have an increased risk of future positive results, and finally sending an alert by letter or email to the farmers of those herds. This approach requires selection of a cut-off value, π*_c_*, to discriminate between herds with high and low risks of future positive results. The choice of this cut-off value needs to be based on careful judgement of the relationship between the cut-off value, the proportion of herds that will receive an alert, and the positive and negative predictive values of the alert (Table 4). By increasing the cut-off value, the positive predictive value can be increased, but this comes at the price of a lower proportion of herds receiving an alert and a lower negative predictive value (i.e., a higher proportion of herds that will have future positive results, despite not having received an alert).

An alternative approach would be to provide each farmer with a herd-specific prediction shortly after the results of a herd examination become available. To do so, the coefficients of the explanatory variables in the models should be re-estimated, as the distribution of some of these variables may be different (such as the distribution of the time since the last positive ELISA result). Given that results of herd examinations of participating herds become available at varying time points throughout the year, development of a software tool for the automated calculation of the model predictions shortly after each herd examination would seem to be a prerequisite for implementing such an alternative approach in a cost-efficient manner.

External validation of predictive models is generally preferred above internal validation [14]. In this study, external validation was not feasible as the milk quality assurance program and risk factors, such as province, were specific to the Netherlands. Nevertheless, the approach followed in this study may be applicable to other disease control programs in other cattle populations.

At present, the prediction models developed in this study are not yet operational. Available options will be discussed with the stakeholders and decision-makers involved in the control of paratuberculosis. The practical applications of our models include management of the expectations of farmers, veterinarians, and other stakeholders. Farmers with an increased risk of positive ELISA results may use the model predictions to make informed decisions on additional control measures, such as preventive management measures, to reduce the potential spread of Map in their herds. It remains to be seen whether farmers do consider a risk in the range of 31 to 67% of positive ELISA results (i.e., PVP values in Table 4) sufficiently high to warrant allocation of resources to such preventive management measures. In herds receiving an alert of an increased risk of positive results, environmental sampling may be a suitable approach, at low costs, to segregate herds with false positive alerts and herds with high risks of the spread of Map [51,52,53,54,55].

## 5. Conclusions

The aim of the present study was to develop a predictive model to alert farmers with the preferred Status A in the Dutch milk quality assurance program if they are at an increased risk of positive ELISA results in a subsequent 30-month period. On the basis of the results of this study, we conclude that discrimination of herds with high (52%) and low (17%) risks of positive ELISA results is feasible. This might help Status A farmers with the highest risks of positive results to make better informed decisions regarding the need to take additional control measures to prevent the spread of any undetected Map infections.

## Figures and Tables

**Table 1:** ELISA kits and cut-off values used in the MQAP for individual milk and serum samples.

Sample	S/P	ELISA A ^1^			ELISA B ^2^
		Jan 2006–Aug 2007	Aug 2007–May 2018	May 2018–Dec 2019	Dec 2019–Present
Serum	≤0.90	Negative	Negative	Negative	Negative
	>0.9 and <1.1	Suspect	Suspect	Positive	
	≥1.1 and <1.2	Positive	Positive		
	≥1.2				Positive
Milk	<0.25	Negative	Negative	Negative	Negative
	≥0.25 and <1.0	Positive			
	≥1.0 and <1.1		Positive	Positive	
	≥1.1				Positive

^1^ IDEXX Paratuberculosis Screening Ab Test; ^2^ ID Screen Paratuberculosis Indirect Screening Test (IDvet).

**Table 2:** Logistic regression model ^†^ for the prediction of at least one ELISA result with an S/P ≥1.0 between January 2016 and June 2018 (training dataset). Data of dairy herds with Status A in the Dutch milk quality assurance program for paratuberculosis on 1 January 2016 (training dataset: 8566 herds) and on 1 January 2019 (validation dataset: 8026 herds).

Variable	Level	Training Dataset						Validation Dataset
		Number of Herds	β (S.E.)	*p* (LR Test)	*p* (Wald)	OR (95% CI)	Adjusted β in Predictive Model	Number of Herds
Province	Gelderland	1504	Reference	<0.001				1408
	Drenthe	404	0.91 (0.13)		<0.001	2.48 (1.91; 3.23)	0.89	379
	Flevoland	139	0.51 (0.21)		0.016	1.66 (1.10; 2.51)	0.50	131
	Fryslân	1202	0.90 (0.10)		<0.001	2.46 (2.01; 3.00)	0.88	1145
	Groningen	375	0.74 (0.14)		<0.001	2.09 (1.58; 2.75)	0.72	374
	Limburg	297	0.18 (0.18)		0.317	1.20 (0.84; 1.72)	0.18	256
	Noord Brabant	1343	0.34 (0.10)		0.001	1.41 (1.15; 1.72)	0.34	1179
	Noord Holland	427	0.30 (0.14)		0.037	1.34 (1.02; 1.78)	0.29	409
	Overijssel	1664	0.41 (0.10)		<0.001	1.51 (1.24; 1.83)	0.40	1588
	Utrecht	541	0.67 (0.13)		<0.001	1.95 (1.51; 2.52)	0.66	500
	Zuid Holland	565	0.30 (0.14)		0.032	1.35 (1.03; 1.77)	0.29	554
	Zeeland	105	0.29 (0.25)		0.242	1.34 (0.82; 2.19)	0.29	103
Soil type	Sand	4780	Reference	0.002				4404
	Clay	1467	0.29 (0.09)		0.001	1.33 (1.13; 1.58)	0.28	1414
	Bog	1050	0.29 (0.09)		0.002	1.34 (1.11; 1.61)	0.29	1014
	Sandy loam	1133	0.17 (0.09)		0.056	1.19 (1.00; 1.41)	0.17	1071
	Other	136	0.42 (0.23)		0.073	1.52 (0.96; 2.40)	0.41	123
Number of adult cattle		8566	0.0048 (0.0005)	<0.001	<0.001	1.01 (1.00; 1.01)	0.0047	8026
Time since last ELISA result with S/P ≥1.0 (days)	≤730	1587	1.20 (0.07)		<0.001	3.32 (2.87; 3.84)	1.18	1413
	>730 and ≤1460	1492	1.41 (0.07)		<0.001	4.08 (3.55; 4.69)	1.38	1329
	>1460 and ≤2190	718	0.79 (0.10)		<0.001	2.19 (1.82; 2.64)	0.77	859
	>2190 and ≤2920	453	0.44 (0.12)		<0.001	1.55 (1.22; 1.97)	0.43	501
	> 2920 or never	4316	Reference	<0.001				3924
Proportion of cattle born elsewhere	0	3778	Reference	<0.001				3720
	>0 and ≤0.05	2551	0.14 (0.06)		0.026	1.15 (1.02; 1.31)	0.14	2112
	>0.05 and ≤1.0	2237	0.34 (0.07)		<0.001	1.41 (1.24; 1.60)	0.34	2194
95th percentile of S/P		8566	2.38 (0.24)	<0.001	<0.001	10.8 (6.71; 17.5)	2.34	8026
Intercept		8566	−3.32 (0.11)		<0.001		−3.28	8026

^†^ −2 log likelihood = 8707.2; Nagelkerke, R^2^ = 0.21; Hosmer–Lemeshow test, χ^2^ = 7.05, df = 8, *p* = 0.531.

**Table 3:** Logistic regression model ^†^ for the prediction of at least two ELISA results with an S/P ≥1.0 between January 2016 and June 2018 (training dataset). Data of dairy herds with Status A in the Dutch milk quality assurance program for paratuberculosis on 1 January 2016 (training dataset: 8566 herds) and on 1 January 2019 (validation dataset: 8026 herds).

Variable	Level	Training Dataset						Validation Dataset
		Number of Herds	β (S.E.)	*p* (LR Test)	*p* (Wald)	OR (95% CI)	Adjusted β in Predictive Model	Number of Herds
Province	Gelderland	1504	Reference	<0.001				1408
	Drenthe	404	0.93 (0.18)		0.000	2.54 (1.80; 3.57)	0.91	379
	Flevoland	139	0.34 (0.27)		0.208	1.41 (0.83; 2.40)	0.34	131
	Fryslân	1202	0.96 (0.14)		0.000	2.60 (1.99; 3.40)	0.94	1145
	Groningen	375	0.65 (0.18)		0.000	1.92 (1.34; 2.74)	0.64	374
	Limburg	297	0.10 (0.26)		0.709	1.10 (0.66; 1.83)	0.09	256
	Noord Brabant	1343	0.33 (0.15)		0.021	1.40 (1.05; 1.86)	0.33	1179
	Noord Holland	427	0.29 (0.19)		0.132	1.33 (0.92; 1.94)	0.28	409
	Overijssel	1664	0.42 (0.14)		0.003	1.53 (1.16; 2.01)	0.41	1588
	Utrecht	541	0.56 (0.18)		0.002	1.75 (1.23; 2.49)	0.55	500
	Zuid Holland	565	0.31 (0.19)		0.102	1.37 (0.94; 1.99)	0.31	554
	Zeeland	105	−0.28 (0.39)		0.474	0.76 (0.35; 1.62)	−0.27	103
Soil type	Sand	4780	Reference	0.016				4404
	Clay	1467	0.32 (0.11)		0.004	1.37 (1.11; 1.70)	0.31	1414
	Bog	1050	0.15 (0.12)		0.227	1.16 (0.91; 1.46)	0.14	1014
	Sandy loam	1133	0.19 (0.12)		0.100	1.21 (0.96; 1.53)	0.19	1071
	Other	136	0.64 (0.30)		0.033	1.90 (1.05; 3.43)	0.63	123
Number of adult cattle		8566	0.01 (0.00)	<0.001	<0.001	1.01 (1.00; 1.01)		8026
Time since last ELISA result with S/P ≥1.0 (days)	≤730	1587	1.74 (0.11)		<0.001	5.68 (4.60; 7.01)	1.70	1413
	>730 and ≤1460	1492	1.87 (0.10)		<0.001	6.46 (5.26; 7.93)	1.83	1329
	>1460 and ≤2190	718	1.33 (0.13)		<0.001	3.79 (2.92; 4.92)	1.30	859
	>2190 and ≤2920	453	0.81 (0.18)		<0.001	2.24 (1.57; 3.20)	0.79	501
	>2920 or never	4316	Reference	<0.001			1.70	3924
Proportion of cattle born elsewhere	0	3778	Reference	<0.001				3720
	>0 and ≤0.05	2551	0.20 (0.09)		0.019	1.22 (1.03; 1.45)	0.20	2112
	>0.05 and ≤1.0	2237	0.33 (0.09)		<0.001	1.39 (1.18; 1.65)	0.32	2194
95th percentile of S/P		8566	2.52 (0.27)	<0.001	<0.001	12.4 (7.28; 21.1)	2.46	8026
Intercept		8566	−4.83 (0.16)		<0.001		−4.77	8026

^†^ −2 log likelihood = 5545.6; Nagelkerke, R^2^ = 0.23; Hosmer–Lemeshow test, χ^2^ = 7.11, df = 8, *p* = 0.525.

**Table 4:** Proportions of herds with predicted probabilities larger than the cut-off value, positive predictive values (PVP), and negative predictive values (PVP) at various cut-off values π_c_ of the predicted probability π of at least one or at least two sample(s) with an S/P ≥1.0. Data of 8566 dairy herds with Status A on 1 January 2016 (training dataset), and 8026 dairy herds with Status A on 1 January 2019 (validation dataset). Results discussed as examples in Section 3.3 and Section 3.4 are printed in bold.

Predictive Model	Cut-Off Predicted Probability *π_c_*	Training Dataset						Validation Dataset				
		Proportion of Herds with Predicted Probability > Cut-Off *π_c_*	Se	Sp	PVP	PVN		Proportion of Herds with Predicted Probability > Cut-Off *π_c_*	Se	Sp	PVP	PVN
≥1 sample with S/P ≥1.0	0.30	0.39	0.67	0.71	0.47	0.85		0.39	0.69	0.72	0.46	0.87
	0.35	0.32	0.59	0.77	0.49	0.83		0.32	0.61	0.78	0.49	0.85
	**0.40**	**0.25**	**0.48**	**0.83**	**0.52**	**0.81**		**0.25**	**0.51**	**0.84**	**0.52**	**0.83**
	0.45	0.19	0.38	0.88	0.56	0.79		0.18	0.39	0.89	0.54	0.81
	0.50	0.13	0.29	0.93	0.60	0.78		0.13	0.28	0.92	0.55	0.79
	0.55	0.08	0.20	0.96	0.64	0.76		0.08	0.19	0.96	0.61	0.78
	0.60	0.05	0.12	0.98	0.67	0.75		0.05	0.12	0.98	0.67	0.77
≥2 samples with S/P ≥1.0	**0.20**	**0.26**	**0.61**	**0.79**	**0.31**	**0.93**		**0.25**	**0.60**	**0.80**	**0.31**	**0.93**
	0.25	0.17	0.46	0.87	0.34	0.91		0.17	0.44	0.87	0.34	0.91
	0.30	0.11	0.33	0.92	0.40	0.90		0.10	0.30	0.93	0.37	0.90
	0.35	0.06	0.21	0.96	0.45	0.89		0.06	0.20	0.96	0.43	0.89
	0.40	0.04	0.15	0.98	0.50	0.88		0.03	0.13	0.98	0.51	0.88
	0.45	0.02	0.10	0.99	0.53	0.88		0.02	0.08	0.99	0.56	0.88
	0.50	0.01	0.06	0.99	0.58	0.88		0.01	0.05	0.99	0.61	0.88
	0.55	0.01	0.04	1.00	0.60	0.87		0.01	0.03	1.00	0.59	0.87
	0.60	0.00	0.02	1.00	0.62	0.87		0.00	0.02	1.00	0.61	0.87

## Data Availability

The herds or groups of herds included in this study may be identifiable from their unique combination of explanatory variables. Therefore, the datasets analyzed in this study cannot, and will not, be made available to readers.
